# Comparison and improvement of algorithms for computing minimal cut sets

**DOI:** 10.1186/1471-2105-14-318

**Published:** 2013-11-06

**Authors:** Christian Jungreuthmayer, Govind Nair, Steffen Klamt, Jürgen Zanghellini

**Affiliations:** 1 Austrian Centre of Industrial Biotechnology, Vienna, Austria; 2 Department of Biotechnology, University of Natural Resources and Life Sciences, Vienna, Austria; 3 Max Planck Institute for Dynamics of Complex Technical Systems, Magdeburg, Germany

**Keywords:** Metabolic network analysis, Elementary modes, Minimal cut sets, Knockout strategies, Integer programming, Berge’s algorithm

## Abstract

**Background:**

Constrained minimal cut sets (cMCSs) have recently been introduced as a framework to enumerate minimal genetic intervention strategies for targeted optimization of metabolic networks. Two different algorithmic schemes (adapted Berge algorithm and binary integer programming) have been proposed to compute cMCSs from elementary modes. However, in their original formulation both algorithms are not fully comparable.

**Results:**

Here we show that by a small extension to the integer program both methods become equivalent. Furthermore, based on well-known preprocessing procedures for integer programming we present efficient preprocessing steps which can be used for both algorithms. We then benchmark the numerical performance of the algorithms in several realistic medium-scale metabolic models. The benchmark calculations reveal (i) that these preprocessing steps can lead to an enormous speed-up under both algorithms, and (ii) that the adapted Berge algorithm outperforms the binary integer approach.

**Conclusions:**

Generally, both of our new implementations are by at least one order of magnitude faster than other currently available implementations.

## Background

The aim of metabolic engineering is to (re-)allocate available cellular resources in order to induce/stimulate cells to produce substances of interest. For instance, by redirecting intracellular carbon fluxes, product yields can be boosted and optimized [1,2]. However, the identification of engineering targets is not straight-forward as cellular metabolism is a highly interconnected and regulated system of reactions. Consequently, naïve interventions sometimes are ineffective or worse, adversely affect other, even quite distant cellular functions. To deal with the complex interactions in cellular metabolism and to identify promising engineering targets several *in silico* approaches have been developed [3-9]. Here we are particularly concerned with two algorithms [10,11], which are based on *elementary mode* (EM) analysis [12,13] and eventually compute intervention strategies as *minimal cut sets*.

EM analysis was successfully used to identify engineering targets for the production of amino acids [14], biofuels [15,16], and secondary metabolites [17] in various organisms from *C. glutamicum*[14] to *E. coli*[15,16] to *S. cerevisiae*[18] to *A. niger*[19]. In fact, EM analysis is ideally suited for metabolic engineering [20,21] as it allows for an unambiguous and unbiased decomposition of the analyzed network into inseparable, biologically meaningful steady-state pathways. Any intracellular flux distribution can be represented as a properly weighted combination of these EMs. Thus, the full set of EMs describes all possible steady-state functions. Conversely, the cell’s metabolic capabilities can be restricted if EMs are removed from the network. To remove an EM from the network it suffices to delete at least one contributing reaction [12,13]. However, as each reaction supports more than one EM, other network functionality will be affected, too. Now the question may be phrased as an optimization problem. The task is to find a minimal intervention strategy, which removes all unwanted functionality from the network while, at the same time, keeps desirable network properties.

Recently, Hädicke and Klamt [11] introduced the concept of constrained minimal cut sets (cMCSs) to predict suitable minimal intervention strategies for a given design criterion. They also presented an algorithm (adapted Berge algorithm [11]; see also [22,23]) by which cMCSs can be computed from EMs. Jungreuthmayer and Zanghellini [10] conceived an alternative method to compute cMCSs by solving a binary integer program (BIP) over the EMs.

By adapting the BIP originally presented in [10] we first demonstrate that both algorithms deliver indeed equivalent results. Inspired by the theory of integer programming, we then develop efficient preprocessing procedures, which allow both methods to handle problems with hundreds of millions of EMs. Finally, by computing intervention strategies in several realistic networks, we benchmark and compare the computational performance of both algorithms.

## Methods

EMs are an unbiased way to characterize metabolic networks. An EM is defined by three properties [12,13]: (i) it is a non-trivial, steady state flux distribution through the network, (ii) it obeys all thermodynamic constraints on reaction reversibilities, and (iii) no subset of an EM exists which also is an admissible flux distribution and obeys (i) and (ii). By this definition, an EM is a minimal, biologically meaningful, steady-state pathway through a network. An EM can be represented as a (flux) vector or by the set of active reactions in the EM. Herein we will mainly use the latter.

In the following we assume that all EMs are known. Several tools to calculate EMs are freely available [24-27].

### cMCS theory

Hädicke and Klamt [11] defined cMCSs as solutions *I* of an intervention problem

(1) I=I(T,D,n).

Here, ***D*** and ***T*** denote sets of desired and target modes, respectively. The latter contains all EMs, which need to be removed from the network. The former contains all EMs with favorable functionality. An intervention *I* will be a set of reactions that are deleted (knocked-out) in the network. An EM is hit (and becomes inoperable) if at least one reaction of *I* is part of the EM. The variable *n* denotes the minimum number of desired EMs, which have to “survive” the intervention. For a given intervention *I*, we collect all the surviving desired modes in the set ***D***_*I*_.

A proper solution *I* of equation (1) is a set of reactions obeying two conditions: First, the removal of the reactions in *I* will delete the complete target set, ***T***, from the network

(2) t∩I≠∅∀t∈T,

and no subset of *I* will do so. This is the MCS property. To be a *constrained* MCS the intervention *I* will keep at least *n* desirable EMs unaffected, i.e.

(3) $$|D_{I}|\geq \mathrm{n.}$$

As each EM represents a unique pathway through a network, removing it from the network means to block that path, which is easily doable by deleting at least one contributing reaction. Thus, to meet condition (2), the task is to find a (minimal) hitting set such that all pathways in ***T*** are blocked [see equation (2)]. Mathematically, this problem is also known as dualization of a (hyper-)graph, a fundamental problem in discrete mathematics [28]. Several algorithms for calculating hitting sets are available, of which the Berge algorithm [22] has been shown to perform favorably for the problems considered herein [23]. However, minimal hitting sets ensure only that all target modes are hit but do not per se ensure the constraint (3), i.e. the survival of *n* desired modes.

A simple strategy to fulfill equation (2) in combination with the constraint in equation (3), is to first calculate all possible minimal hitting sets and then, in a second step, to only select those solutions which also obey equation (3). However, the computational performance can be optimized if the constraint equation (3) is checked “on the fly”, leading to the adapted Berge algorithm presented in [11].

A pseudo-code of the adapted Berge algorithm can be found in [11], in the following we give a small example to explain basic principles of the Berge algorithm. Consider a hypergraph with hyperedges *ε*_1_={*a*,*b*} and *ε*_2_={*a*,*c*} (in our application, *ε*_1_ and *ε*_2_ would represent target EMs). The algorithm finds first all minimal hitting sets (cut sets) for the first edge, i.e. *γ*_1_={*a*} and *γ*_2_={*b*}. It then adds the next edge, *ε*_2_, and checks whether the already calculated cut sets are also cut sets for the current edge. Since *γ*_1_ is hitting *ε*_2_, *γ*_1_ is kept unchanged. However, *γ*_2_ is not a cut set for *ε*_2_ and, thus, is removed from the list of cut sets. Instead, two new cut sets are created by individually adding each element of *ε*_2_ to *γ*_2_, i.e. *γ*_3_={*b*,*a*} and *γ*_4_={*b*,*c*}. To guarantee minimality the algorithm checks if a newly created cut set is a superset of an already existing one. That is, *γ*_3_ gets removed from the set of cut sets as it is a superset of *γ*_1_. Next, a new edge is added to the system and the calculation cycle starts over. Execution stops when all hyperedges are processed. To account for the intervention problem and accelerate the classic algorithm, Hädicke and Klamt suggested to first check if a newly generated cut set is consistent with the constraint (3) and only then check its minimality against all previously calculated cut sets [11]. This modification leads to the adapted Berge algorithm [11] which will be used in the following.

### cMCSs can be formulated as a BIP

In a recent paper [10] we showed that if | ***D*** |=*n* then the intervention *I*=*I*(***T***, ***D***,*n*) is representable as a BIP. However, even the general problem that at least *n* out of | ***D*** | modes need to survive the intervention can be formulated as a BIP.

Let ***e*** be an EM of a metabolic network with *m* reactions, fulfilling the steady state condition, and ***b*** = ***b*** (***e***) its binary representation,

(4) $$b^{i}:=b^{i}(e^{i})=\left\{\begin{array}{cc} 1 & \text{if}\;e^{i}\neq 0\\ 0 & \text{if}\;e^{i}=0 \end{array}\right),\;i=\{1,\ldots ,m\}.$$

*b*^*i*^ indicates whether reaction *i* is part of the EM ***e***.

A solution ***x*** to equation (1) can be found by solving the following BIP:

(5a) $$\begin{array}{ll} \;\text{max}\;\;||x|| & \; \end{array}$$

(5b) $$\begin{array}{ll} \;\text{s.t.}\;\;b_{d}^{T}x\geq ||b_{d}||y^{d},\; & \;d\in \{1,\mathrm{..},|D|\},\; \end{array}$$

(5c) $$\begin{array}{ll} \;b_{d}^{T}x\leq ||b_{d}||(1+y^{d})-1,\; & \; \end{array}$$

(5d) $$\begin{array}{ll} \;b_{t}^{T}x\leq ||b_{t}||-1,\; & \;t\in \{1,\mathrm{..},|T|\},\; \end{array}$$

(5e) $$\begin{array}{ll} \;||y||\geq n,\; & \; \end{array}$$

(5f) $$\begin{array}{ll} \;x={\left(x^{1},\ldots ,x^{m}\right)}^{T},\; & \;x^{i}\in \{0,1\}\forall i,\; \end{array}$$

(5g) $$\begin{array}{ll} \;y={\left(y^{1},\ldots ,y^{\left|D\right|}\right)}^{T},\; & \;y^{i}\in \{0,1\}\forall \mathrm{i.}\; \end{array}$$

Here we used the indices *d* and *t* as a reminder that the EM vectors, ***b***_*i*_, are elements of the sets ***D*** and ***T***, respectively. The solution vector, ***x***, is the binary representation of a single cMCS, where *x*^*i*^=0 marks reactions which get deleted, while *x*^*i*^=1 stands for reactions that remain unaffected by the genetic intervention. The elements of the binary, auxiliary vector, ***y***, indicate whether or not a desirable mode survives the intervention (1 and 0, respectively). Note that our notation uses *superscripts* to denote *coordinates* of vectors and *subscripts* to denote different *vectors*. Finally, $||x||:=\overset{m}{\underset{i=1}{\sum }}x^{i}$ represents the multilinear norm of ***x***.

Suppose *y*^*d*^=1, then equation (5c) always holds and can be omitted. Equation (5b) requires that $x^{i}\geq b_{d}^{i}$, ∀*i*∈{1,…,*m*}. Only then the product of $b_{d}^{T}$ and ***x*** is equal to the norm of ***b***_*d*_. Thus, ***b***_*d*_ is included in the final design. In contrast to ***b***_*d*_, ***b***_*t*_ will be removed from the network as equation (5d) requires that the product $b_{t}^{T}x$ is smaller than the || ***b***_*t*_||. This is the case only if at least one reaction in ***b***_*t*_ is deleted. Except for equation (5e), the systems of equations in this case resembles the BIP problem presented in Jungreuthmayer and Zanghellini [10].

If *y*^*d*^=0, then equation (5b) is ineffective. Equation (5c) however simplifies into a “kill constraint”, thus eliminating ***b***_*d*_ from the surviving modes.

The binary auxiliary variables ***y*** =(*y*^1^,…,*y*^| ***D*** |^)^T^ were introduced to guarantee that at least *n* out of | ***D*** | modes survive the intervention. In both cases || ***y*** || counts the number of surviving desired modes, and equation (5e) makes sure that at least *n* desired modes survive the intervention.

Alternative MCSs may be calculated by excluding existing solutions ***x***_*j*_ by adding the following constraints [10] to the set of equations (5a)-(5g):

(6a) $$\begin{array}{l} \;x_{j}^{T}x\leq ||x_{j}||-1,\; \end{array}$$

(6b) $$\begin{array}{l} {\left[1-x_{j}\right]}^{T}x\geq 1,\; \end{array}$$

where we used ***1*** to denote an all-one-vector. Equation (6a) guarantees that new solutions are found in subsequent steps, whereas equation (6b) prevents the calculation of solutions that are supersets of already existing solutions. Note that the term ***1***−***x***_*j*_ represents the binary complement of ***x***_*j*_.

The number of constraints added to the BIP can almost be cut in half (in fact, *n*/2−1) by keeping in mind that the norm of the current solution ***x***_*j*_ will never be larger than the previous optimum ***x***_*j*−1_. To sequentially calculate all MCSs the full BIP reads

(7a) $$\begin{array}{ll} \;\text{max}\;||x_{j}|| & \; \end{array}$$

(7b) $$\begin{array}{ll} \;\text{s.t.}\;b_{d}^{T}x_{j}\geq ||b_{d}||y^{d},\; & \;d\in \{1,\mathrm{..},|D|\},\; \end{array}$$

(7c) $$\begin{array}{ll} \;b_{d}^{T}x_{j}\leq ||b_{d}||(1+y^{d})-1,\; & \; \end{array}$$

(7d) $$\begin{array}{ll} \;b_{t}^{T}x_{j}\geq ||b_{t}||-1,\; & \;t\in \{1,\mathrm{..},|T|\},\; \end{array}$$

(7e) $$\begin{array}{ll} \;||x_{j}||\leq ||x_{j-1}||,\; & \;||x_{0}||=m,\; \end{array}$$

(7f) $$\begin{array}{ll} \;{\left[\;1\;-\;x_{k}\right]}^{T}\;x_{j}\geq 1,\; & \;k\in \{0,\mathrm{..},j\;-\;1\},\; \end{array}$$

(7g) $$\begin{array}{ll} \;||y||\geq n,\; & \; \end{array}$$

(7h) $$\begin{array}{ll} \;x_{j}={\left(x_{j}^{1},\ldots ,x_{j}^{m}\right)}^{T},\; & \;x^{i}\in \{0,1\}\forall i,\; \end{array}$$

(7i) $$\begin{array}{ll} \;y={\left(y^{1},\ldots ,y^{\left|D\right|}\right)}^{T},\; & \;y^{i}\in \{0,1\}\forall \mathrm{i.}\; \end{array}$$

If iteratively applied, the BIP in equation (7) returns all MCSs, ***x***_*j*_, sorted in increasing order of deletions. Note that although the constraint in equation (7e) is redundant, it significantly enhances the computational performance of the BIP solver.

### Preprocessing methods

Mathematically, BIPs are classified as NP-hard problems. However, extensive research has focused on improving the formulation of BIPs. The basic idea is to use simple logic rules which turn a BIP into a “better” formulation, which is easier to solve [29]. Standard BIP preprocessing rules essentially fix variables, improve bounds or detect inactive constraints [29].

In the following we will be concerned with standard BIP preprocessing methods to reduce the size of the intervention problem in equation (1) but not with the internal structure of the algorithms. These preprocessing procedures will allow to reduce the size of the intervention problem in equation (1), which can then be solved by the Berge algorithm or a BIP. In the following, by “Berge algorithm” we mean the adapted Berge algorithm reported by Hädicke and Klamt [11] which extends the standard Berge algorithm to compute only minimal hitting sets (cut sets) that comply with the constraint (3) on the desired modes [11].

We assume that all EMs are converted to their binary representation according to equation (4). Furthermore, we split the complete set of EMs in three sets, ***D***, ***N***, and ***T***. Here the neutral set, ***N***, contains all (binary) EMs, which are neither element of ***D*** nor ***T***.

**Step 1.** First, we remove all reactions that are simultaneously zero in all EMs of ***T***. These reactions do not support any EM in ***T***. Deleting them will not remove any unwanted mode.

**Step 2.** Next, essential reactions are identified. If deleting a reaction reduces the number of surviving modes in ***D*** to less than *n* [i.e. violates equation (3)], then this reaction is considered to be essential and cannot be knocked out. A reaction *i* is essential if | ***D*** |−*s*^*i*^<*n*, with $s^{i}=\overset{\left|D\right|}{\underset{j=1}{\sum }}x_{j}^{i}$.

Consider the example in Table 1 with | ***D*** |=5 and *n*=3. *R*1 and *R*7 are essential reactions, as for them | ***D*** |−*s*^*i*^=5−3=2<3=*n*, which indicates that knocking out *R*1 or *R*7 will kill more desirable modes than allowed. We note that if | ***D*** |=*n*, all active reactions are essential.

**Table 1:** Example of determining essential reactions

	**R1**	**R2**	**R3**	**R4**	**R5**	**R6**	**R7**	**R8**
$d_{1}^{T}$	0	1	0	0	1	0	1	0
$d_{2}^{T}$	0	0	0	0	0	1	0	0
$d_{3}^{T}$	1	0	1	0	1	0	0	0
$d_{4}^{T}$	1	1	0	0	0	0	1	0
$d_{5}^{T}$	1	0	1	0	0	0	1	0
***s***	3	2	2	0	2	1	3	0

In general, the more essential reactions we find, the more the system can be reduced. Consequently, it is beneficial if *n* is large (ideally *n*=| ***D*** |), as this results in the maximum number of essential reactions. Removing all essential reactions from the system is a critical step that opens the possibility to apply several other preprocessing procedures.

The removal of all essential reactions results in an important change of the system. By definition EMs are non-decomposable, i.e. an EM is not a subset of any other EM. However, if the essential reactions are removed then the residual EMs may become subsets or duplicates of other modes. Hence, the next step is to find all duplicate modes in ***T*** and to remove them from the system.

**Step 3.** Next, we screen ***T*** to find and remove residual EMs that are supersets of other residual EMs in ***T***. Consider the target set (of residual EMs), ***T***, shown in Table 2. The modes are sorted in order of ascending norm. The example illustrates that mode ***t***_1_ can be removed by knocking out reaction *R*2. However, knocking out reaction R2 also kills ***t***_2_, as ***t***_2_ is a superset of ***t***_1_.

**Table 2:** Set of (residual) target modes before and after subset-superset elimination

	**R1**	**R2**	**R3**	**R4**	**R5**	**R6**	**R7**	**R8**	**||** ***t***_***i***_**||**	
$t_{1}^{T}$	0	1	0	0	0	0	0	0	1	
$t_{2}^{T}$	0	1	0	0	1	0	0	0	2	*
$t_{3}^{T}$	0	0	0	1	0	0	1	0	2	
$t_{4}^{T}$	1	0	0	1	0	0	1	0	3	*
$t_{5}^{T}$	0	0	0	1	0	0	1	1	3	*
$t_{6}^{T}$	1	0	1	0	0	0	1	1	3	

Modes which are removed during the preprocessing, are marked by *. Note that the residual target modes ***t***_1_, …, ***t***_6_ are no longer EMs, as they have already gone through step 1 and 2 of our preprocessing procedure.

The same procedure can be applied to the other modes as well. Mode ***t***_3_ has a norm of 2 and is killed either by knocking out reaction R4 or reaction R7. As both reactions are part of ***t***_4_ and ***t***_5_, they are certainly removed if mode ***t***_3_ is killed.

**Step 4.** In a final preprocessing step, we remove duplicate reactions across all EMs in both sets, ***D*** and ***T***. Using the illustration in Table 2, this would mean that we remove duplicate columns. Note that this step is most effective after all supersets were removed. For instance, in Table 2 columns R1, R3 and R8 are identical only if ***t***_2_, ***t***_4_, and ***t***_5_ are removed. In this step it is not possible to analyze ***D*** and ***T*** separately. Reactions need to be identical in both sets, ***D*** and ***T***, in order to be removed.

### Implementation

We implemented the BIP algorithm in C using Gurobi Optimizer 5.0, http://www.gurobi.com/ for solving the BIP problem. The adapted Berge algorithm was implemented in C. The software is available from the authors on request. The simulations were all carried out on an Intel Xeon CPU X5650 @ 2.67GHz under a Linux operating system.

### Test cases

**Figure 1:**
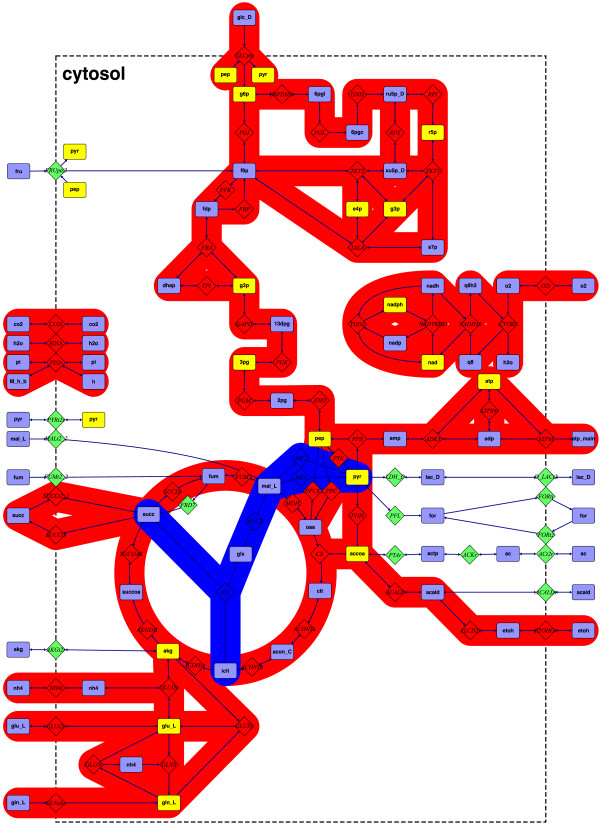
**Overview of the different *E. coli* models.** For simplicity we only show pathways. Cofactors etc. are suppressed. Metabolites contributing to the biomass are depicted in yellow. Pathways included in the E2 model are indicated in red. E1 contains the red and blue pathways only, while E0 [30] incloses all reactions, including the non-colored pathways. A detailed listing of all models may be found in the Additional file 1.

We used the *E. coli* core model, E0, [30] and two smaller models, E1 and E2, which were derived from the E0 model by removing several reactions. Compared to the E0 model, glucose was considered as the only carbon substrate for E1 and exchange of *α*-ketoglutarate, acetaldehyde, acetate, formate, lactate, and pyruvate was not allowed. In addition to these modifications we also removed the glyoxylate shunt and the (NAD and NADP dependent) malic enzymes to obtain the E2 model from E1. All three models are illustrated in Figure 1. Their main topological properties are summarized in Table 3. A list of reactions for these models may be found in the (Additional file 1).

**Table 3:** **Topological properties of the *E. coli* models used**

	**E2**	**E1**	**E0**
Metabolites	63	64	74
- Internal	49	50	52
- External	14	14	22
Reactions	60	64	75
- Irreversible	26	30	36
- Reversible	34	34	39
Elementary modes	55,666	485,169	124,341,216

**Figure 2:**
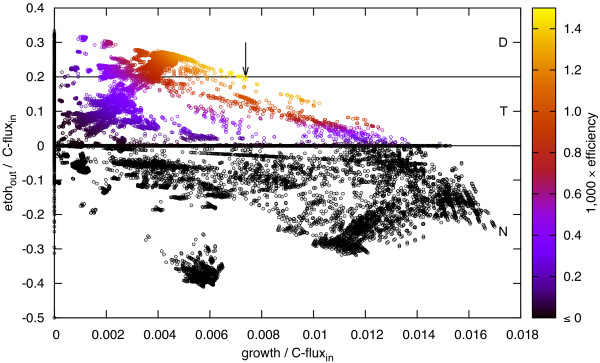
**Phenotypic plot of all EMs in E2. Flux values are normalized to the total carbon influx (C- flux**_**in**_**).** EMs are color coded with respect to the modes’ efficiency, defined as the product between normalized growth and normalized ethanol secretion (etoh_out_). The most efficient EM is marked by an arrow. The areas D, N, and T indicate the corresponding sets of EMs for the intervention problem *I*_*n*_(***T***,***D***,*n*) with *n *∈ {1,…,*n*_max_}.

To test the numerical efficiency of the implemented MCS algorithms we set up the following intervention problems: We first identify the most efficient EMs in all models. Efficiency is defined as the product between growth rate and ethanol secretion. Next, we classify all EMs to be desirable, whose ethanol secretion is larger or equal than the excretion of the most efficient EMs. Targets are all other modes that do not utilize ethanol. Modes which take up ethanol (negative secretion) are considered neutral, as ethanol uptake is repressed in the presence of glucose in the growth medium [31]. Therefore these modes do not need to be targeted. In Figure 2 we illustrate the intervention problem and the choice of ***D***, ***N***, and ***T*** for the E2 model. The major properties of the design criteria for the different *E. coli* models are listed in Table 4.

**Table 4:** **Cardinalities for the sets involved in the intervention problem *I***_***n***_**(*T*,*D*,*n*) with *n∈ *{*1*,*…*,*n***_**max**_**}**

	** E2**		** E1**	
| ***D*** |	5,132	(9%)	46,254	(10%)
| ***N*** |	18,447	(33%)	217,877	(45%)
| ***T*** |	32,087	(58%)	221,038	(45%)
*n*_max_	1,120	(2%)	11,436	(2%)

*n*_max_ denotes the maximum number of “surviving” modes for a given set of ***T*** and ***D***. That is, for *n*>*n*_max_ the intervention *I*_*n*_ is infeasible. Numbers in brackets give the cardinality in percent of the total number of EMs.

## Results

### Berge algorithm outperforms the BIP

**Figure 3:**
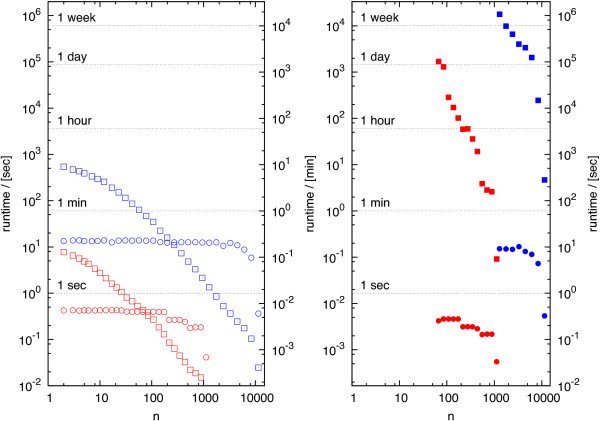
**Runtime for the Berge algorithm (left panel) and the BIP (right panel) for the models E2 (red) and E1 (blue), respectively.** The runtimes for preprocessing (circles) and calculating MCSs are plotted as function of *n*. *n* is the lower bound of desired EMs, which must survive the intervention. Table 4 gives the size of the intervention problem for both models. As the trend is clearly visible, we did not evaluate the BIP for *n *< 80.

Figure 3 shows the computation time to calculate all MCSs using either method as a function of the minimally required number *n* of surviving desired EMs. We used the design criteria outlined above. At *n*=2 we found 81,168 and 441,095 MCSs in E2 and E1, respectively. (The number of MCSs as function of *n* may be found in Additional file 2: Figure S1.) In all tested situations the adapted Berge algorithm clearly outperforms the BIP. Even in the most demanding case (*n*=2), the Berge algorithm calculated all MCSs in E1 in less than 10 min. On the other hand, it already took the BIP 22 hours to calculate all 331 MCSs for *n*=85 in the smaller E2 model. In the same situation the Berge implementation finished in 0.4 sec.

It is interesting to observe that over a wide range of values for *n* the runtime in both methods changes according to a power law (see Figure 3). However, only for the Berge algorithm the exponent remained approximately constant in both test cases.

Preprocessing-times are essentially independent of *n* and only scale with the total number of processed EMs. For cases with very few MCSs (see Additional file 2: Figure S1) the Berge algorithm took even less time than the preprocessing.

### Preprocessing reduces overall computation time

To test the impact of our preprocessing procedures we set up identical intervention problems for all models. That is, we solved *I*_0_=*I*(***T***_0_, ***D***,*n*), *I*_1_=*I*(***T***_1_, ***D***,*n*), and *I*_2_=*I*(***T***_2_, ***D***,*n*), where we used the indices 0, 1, and 2 to denote the dependence on the models E0, E1, and E2, respectively. We used identical sets of desired EMs in all models, i.e. ***D***_0_= ***D***_1_= ***D***_2_= ***D*** and *n*_0_=*n*_1_=*n*_2_=*n*. ***T***_*i*_,*i*∈{0,1,2} consisted of all EMs not contained in ***D***. Values for ***D***, *n*, ***T***_*i*_ and the runtimes for the Berge algorithm in two different cases (*n*/| ***D*** |≈1 and *n*/| ***D*** |≪1) may be found in Table 5.

**Table 5:** **Runtime of the Berge algorithm with and without preprocessing (PP) for two intervention problems *I*(*T*,*D*,*n*) with differing *n***

	**E2**				**E1**				**E0**			
| ***D*** |	489		(0.88%)		489		(0.10%)		489		(0.00%)	
| ***T*** |	55,177		(99.12%)		484,680		(99.90%)		124,340,727		(100.00%)	
				*n*_2_=40; *n*_2_/| ***D*** |=0.08								
MCSs	4				11				44			
Min. deletions	5				8				16			
Max. deletions	5				8				17			
	Without PP		With PP		Without PP		With PP		Without PP		With PP	
System size	60×55,177		13×1		64×484,680		17×3		75×124,340,727		28×6	
Reading EMs (sec)	0.031	(26%)	0.035	(43%)	0.281	(26%)	0.308	(37%)	71.858	(24%)	76.274	(26%)
Preprocessing (sec)	0.078	(65%)	0.044	(55%)	0.717	(65%)	0.495	(60%)	194.328	(64%)	202.539	(69%)
Calculate MCSs (sec)	0.011	(9%)	0.001	(2%)	0.100	(9%)	0.021	(3%)	35.866	(12%)	15.294	(5%)
Total (sec)	0.121	(100%)	0.081	(100%)	1.099	(100%)	0.823	(100%)	302.053	(100%)	294.108	(100%)
				*n*_2_=40; *n*_2_/| ***D*** |=0.08								
MCSs	72				274				1,720			
Min. deletions	5				6				14			
Max. deletions	7				10				19			
	Without PP		With PP		Without PP		With PP		Without PP		With PP	
System size	60×55,177		47×2,295		64×484,680		51×8,664		75×124,340,727		62×321,272	
Reading EMs (sec)	0.031	(10%)	0.033	(25%)	0.277	(7%)	0.308	(20%)	71.084	(3%)	77.771	(5%)
Preprocessing (sec)	0.078	(26%)	0.083	(65%)	0.715	(17%)	1.162	(76%)	193.805	(7%)	1,493.367	(94%)
Calculate MCSs (sec)	0.196	(64%)	0.012	(10%)	3.136	(76%)	0.068	(4%)	2,475.110	(90%)	15.909	(1%)
Total (sec)	0.306	(100%)	0.128	(100%)	4.129	(100%)	1.539	(100%)	2,740.004	(100%)	1,587.053	(100%)

In all cases ***D*** are identical and ***T*** chosen such that it contains all remaining EMs. Note that the columns “without PP” state the runtimes without performing step 1 to 4 of our PP procedures. However, we still sort EMs in ascending order of norm. This is why PP-time is not zero even in the cases without PP. The row “system size” refers to the dimensions of the network, which enters the Berge-algorithm, i.e. after PP.

In the most demanding case (*n*/| ***D*** |≪1), the Berge algorithm with preprocessing identified 1,720 MCSs in less than 30 minutes in the large E0 model with its 124 million EMs (see Table 5). Only 1% of the computation time was used for the Berge algorithm. Ninety-four percent of the computation is spent on preprocessing. After preprocessing the initial system of 124 million EMs was reduced to approximately 300,000 modes. In all tested cases with enhanced preprocessing, reading EMs and preprocessing took at least 90% of the total computation time. We repeated the same simulation without preprocessing. While the total runtime with and without preprocessing is comparable if only a few MCSs are found, the runtime savings in MCS calculation more than outweigh the runtime losses due to preprocessing if many MCSs solve an intervention problem. To emphasize this point we show the total runtime as function of the number of MCSs for Figure 3 in the Additional file 2: Figure S1.

Finally in Table 6 we show several examples from the literature, which can be easily and efficiently solved by either method. As a comparison we have also listed runtimes using the current version (version 2012.1) of CellNetAnalyzer (CNA) [32]. CNA uses a MATLAB implementation of the Berge algorithm. However, its preprocessing capabilities are less developed. That is why both programs, our Berge-algorithm and BIP, outperform CNA in all instances by at least one order of magnitude. Note however that CNA uses a MATLAB script, while our programs are implemented in C. A significant part of the performance difference may therefore be attributed to the slower performance of MATLAB compared to native executables written in C.

**Table 6:** Runtime analysis for the Berge algorithm and BIP using several examples from the literature with different design objectives

							**Runtime (sec)**		
**Organism**	**Objective**	**|** ***D*** **|**	**|** ***T*** **|**	**# MCS**	**Min.** ***Δ***	**Max.** ***Δ***	**Berge**	**BIP**	**CNA**
*E. coli*[16] (anaerobic)	ethanol	12	4,998	1,048	6	9	0.011	0.287	2.83
*E. coli*[16] (aerobic)	ethanol	12	429,264	55,488	11	15	0.883	2.174	547.61
*E. coli*[15] (anaerobic)	isobutanol	7	5,615	760	7	10	0.011	0.233	2.69
*E. coli*[33] (anaerobic)	n-butanol	7	7,341	2,280	7	10	0.015	0.226	3.43

Both algorithms use all preprocessing procedures. For comparison we also use the program package *CellNetAnalyzer*[32] which uses a MATLAB script of the Berge algorithm. (Abbreviations: #MCS, number of MCS; min. *Δ*, minimal number of deletions; max. *Δ*, maximal number of deletions; CNA, CellNetAnalyzer).

### Preprocessing strongly reduces the system size

**Figure 4:**
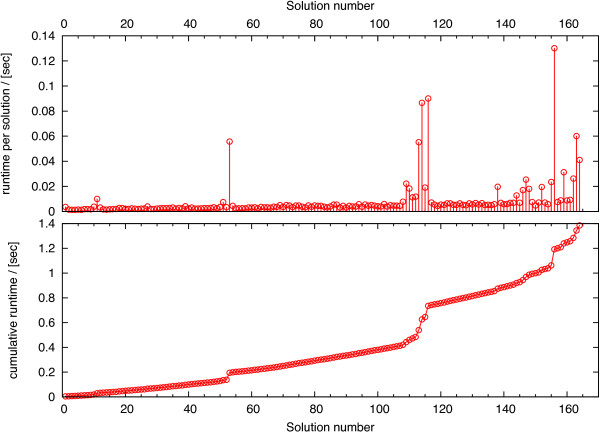
**Computation time of the BIP as function of the number of MCSs for the aerobic*E. coli***[33]**model of Trinh*et al.***[16]**(line number 2 in Table**6**).** The accumulated computation time for the respective models is plotted in the lower panel. The top panel shows the computation time for each solution. Note that it is impossible to calculate the runtime per solution for the Berge algorithm as the algorithm does not allow to continuously output the solution.

In Figure 4 we used a BIP and show the computation time as a function of the number of MCSs for the aerobic *E. coli*[33] model of Trinh *et al.*[16] (see line number 2 in Table 6 for model details). Note that although Table 6 lists 55,488 different MCSs, the BIP (and our Berge algorithm for that matter) only needs to calculate 164 solutions. Due to preprocessing the original network is reduced from 71 reactions and 429,275 EMs to an equivalent system with only 23 columns and 28 rows. This smaller system has 164 MCSs, which are then reconstructed to the full set of MCSs by expanding duplicated columns. A similar observation may also be made in Table 5. In these examples the system size is at least reduced by a factor of 30 (case E2, *n*_2_=40).

Surprisingly, the computation time does not monotonically increase with the number of solutions [i.e. with the number of additional constraints, see equation (7)] but drops dramatically whenever the norm of the solution decreases. Note that a decreasing norm means an increase in the number of required knockouts. In this model the computation time significantly drops after solution number 11, 53, 116, and 156. At these instances the number of required deletions changes from 11 to 12, to 13, to 14, and to 15, respectively. In all these cases the constraint in equation (7e) decreases too and introduces a tighter bound on the system. This allows the solver’s internal preprocessing to more efficiently compress the system, which in turn brings down the computation time. If, however, the norm of the solution does not change then the computation time scales approximately exponentially with the number of MCSs. This behavior is expected as each solution adds a new constrain to the system, which makes it harder to solve.

## Discussion

Recently cMCSs have been introduced to predict optimal intervention strategies in order to achieve an arbitrary metabolic objective [11]. Two algorithmic approaches have been published for their calculation [10,11]. Here we showed that both methods are equivalent. We addressed the numerical efficiency of both methods in typical design problems and found that in terms of runtime the Berge algorithm is superior compared to BIP.

It may appear as a surprise that the Berge algorithm performs so well even for the large cases presented in this study, especially since the Berge method is known for its unfavorable performance in huge networks [34]. However, here we showed that efficient preprocessing can dramatically reduce the size of the networks. The adapted Berge algorithm could then be run on the reduced systems. Apparently, for small systems the Berge algorithm is effective.

The importance of preprocessing in the calculation of MCSs has been stressed earlier [23]. The preprocessing strategies introduced herein focus especially on the additional constraints posed by cMCSs, whereas [23] dealt only with (unconstrained) MCSs. We were able to show that our implementation outperforms the currently available tool for computing (c)MCSs (see Table 6). The performance gain can be attributed to both the improved preprocessing and the efficient implementation in C. Herein we used standard preprocessing routines, which are frequently applied in BIP [29]. Extensive literature on preprocessing in binary and integer programming is available, see for instance Savelsbergh [35] for a good summary of basic ideas. Since cMCSs can be stated as a BIP, these methods are readily adoptable. However, due to the algorithmic complexity of BIP (solving numerous linear optimizations as part of one BIP, etc.), a full enumeration based on BIP seems not be competitive compared to the Berge algorithm (see Figure 3). Rather the usage of BIP preprocessing rules followed by the Berge algorithm to calculate cMCSs is suggested as an optimal computation strategy.

The efficiency of preprocessing is dependent on the imposed design criterion. In the worst case the set of desired modes is empty (***D*** =*∅*) and ***T*** contains all EMs of a network. This situation corresponds to unconstrained MCSs and thus to a full dualization of the hypergraph spanned by the target modes. Except for step 4, none of our preprocessing routines then provides an advantage and other solvers may be more appropriate [34]. However such cases are not relevant in the context of metabolic engineering, where we want to optimize favorable functionality. To fully utilize the potential of preprocessing the ratio *n*/| ***D*** | should be close to one. This means that many essential reactions will be removed from the system, and as a result of that many supersets will be detected, too. However, in practice it may suffice if only a few EMs out of the set of desired modes survive, i.e. *n*/| ***D*** |≪1. Still, preprocessing provides a significant performance gain as indicated in Table 5. The runtime costs of preprocessing will be outweighed by the savings in MCS calculation, if the intervention problem has many solutions. In practice, preprocessing will therefore be favorable, as typical applications have a few thousand solutions (see Table 6).

In our paper [10] we used weights in the objective function of the BIP to take experimental difficulties in the deletion of reactions into account. For instance, some reactions cannot be deleted as they are driven by diffusion, rather than catalyzed by an enzyme. Other reactions, on the other hand, may require the deletion of multiple genes as they are catalyzed by different enzymes in parallel. By an appropriate choice of the weights in the objective function BIP is able to predict the experimentally easiest deletion strategies first [10]. However, in the preprocessing procedures above we did not consider weights in the objective function. Identifying particular solutions in the complete list of MCSs has to be done in a separate post-processing step (for example by appropriately sorting the output, which can be done quite fast). Thus even with an additional post-processing step our implementation of Berge’s algorithm will be faster than BIP. Note however that the integration of regulatory information into the cMCS framework is a unique feature of the BIP approach [10].

Both methods, the Berge algorithm as well as BIP, still show room for computational improvements. In the case of the Berge algorithm the computational bottleneck sits in the filtering of potential MCSs to determine if they are, in fact, true MCSs and not supersets of true MCSs [23]. Generating new MCS-candidates, however, is very quick. Therefore ways of enhancing the superset-filtering procedure will be the scope of future work.

One disadvantage of the Berge algorithm is its inability to predict MCSs continuously during the runtime. During execution all MCSs remain candidate-MCSs. Only upon termination, when the minimality of all candidate MCSs has been checked against each other, candidate-MCSs become MCSs and can be outputted. Thus, even if we were interested in only one solution, the Berge algorithm will – in general – return more than one MCS upon termination. However, other solvers are available [34]. Their adaption for the current situation is the scope of further work.

BIP on the other hand, is able to predict a single solution without the need to enumerate all. In fact, due to the optimization principle only one MCS with a smallest or largest number of deletions can be calculated. In Figure 4 we illustrated the runtime per solution as function of the number of MCSs. The drop in runtime after certain solutions indicates that more advanced preprocessing procedures may further reduce the runtime significantly. In fact, our preprocessing focused on standard procedures like variable fixing. More advanced methods will further reduce the runtime for both the Berge algorithm and the BIP. Additionally we used GUROBI, a commercially available multi-purpose optimization toolbox, to solve the BIP. However, a specialized knapsack solver may potentially boost the performance.

## Conclusions

We predicted minimal metabolic intervention strategies in typical metabolic engineering problems using two different methods (an adapted Berge algorithm and a BIP). We investigated the numerical performance of these approaches. Both methods significantly profited from the enhanced preprocessing procedures developed here. Under the tested conditions, our implementation of Berge’s algorithm performed best even outperforming other, currently available software.

## Abbreviations

BIP: Binary integer program; cMCS: Constrained minimal cut set; CNA: CellNetAnalyzer; EM: Elementary mode; MCS: Minimal cut set; PP: Preprocessing.

## Competing interests

The authors declare that they have no competing interests.

## Authors’ contributions

CJ implemented the algorithms and participated in the design of the study. GN coded the models and helped in running the analysis. SK participated in the design of the study. JZ conceived of the study, and participated in its design and coordination. CJ, SK and JZ wrote the manuscript. All authors read and approved the final manuscript.

## Supplementary Material

Additional file 1 tar gzipped archive of the SBML files for the models E0, E1, and E2. Click here for file

Additional file 2 Total runtime with and without preprocessing for the Berge algorithm. Click here for file
