# Supplementary material for: Comparison and improvement of algorithms for computing minimal cut sets
Source: BMC Bioinformatics. 2013 Nov 6;14:318. doi: 10.1186/1471-2105-14-318 (PMC3882775; doi:10.1186/1471-2105-14-318)
Supplement: Additional file 2 — Total runtime with and without preprocessing for the Berge algorithm. [file 1471-2105-14-318-S2.pdf]

C. Jungreuthmayer *et al.* (2013), Additional File 2

Total runtime with and without preprocessing for the Berge algorithm.

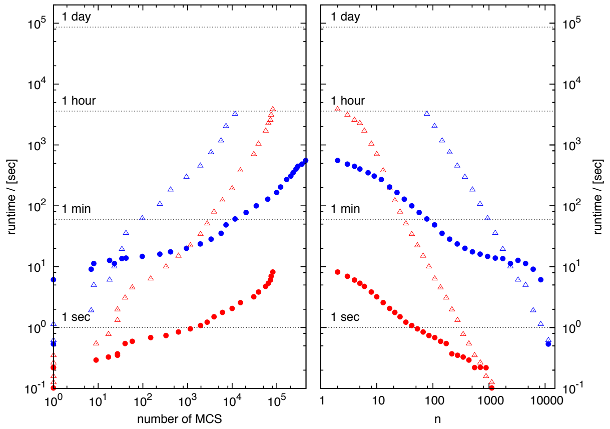

Figure 1: Total runtime with (full circle) and without (open triangle) preprocessing for the Berge algorithm as function of the number of MCS (left panel) for the models E2 (red) and E1 (blue), respectively. The right panel shows the total runtime as function of n to allow for a correlation of the number of solutions with n. All settings are as in Figure 3 of the main text.
